# Histidine Metabolic Pathway Modifies the Relationships Between 6:2 Cl-PFESA Exposure and Preterm Birth

**DOI:** 10.3390/toxics14020142

**Published:** 2026-01-30

**Authors:** Jianping Cong, Chu Chu, Zhitao Zhang, Gaoyuan Sun, Yan Zhang, Aaron M. Qian, Michael G. Vaughn, Sarah Dee Geiger, Kun Zhao, Yunting Zhang, Yang Zhou, Zhihua Yin, Guanghui Dong

**Affiliations:** 1 Shenyang Clinical Medical Research Center for Obstetrics and Gynecology, Liaoning Province Key Laboratory of Assisted Reproduction, Department of Obstetrics and Gynecology, Shenyang Women’s and Children’s Hospital, Shenyang 110011, China; 2 Department of Epidemiology, School of Public Health, China Medical University, Shenyang 110122, China; 3 Joint International Research Laboratory of Environment and Health, Ministry of Education, Guangdong Provincial Engineering Technology Research Center of Environmental Pollution and Health Risk Assessment, Department of Occupational and Environmental Health, School of Public Health, Sun Yat-sen University, Guangzhou 510080, China; 4 Department of Clinical Laboratory, General Hospital of Northern Theater Command, Shenyang 110016, China; 5 Department of Epidemiology and Biostatistics, College for Public Health & Social Justice, Saint Louis University, Saint Louis, MO 63103, USA; 6 School of Social Work, College for Public Health & Social Justice, Saint Louis University, Saint Louis, MO 63103, USA; 7 Department of Kinesiology and Community Health, University of Illinois at Urbana-Champaign, Champaign, IL 61820, USA

**Keywords:** PFAS, 6:2 Cl-PFESA, preterm birth, metabolomics analysis, histidine

## Abstract

**Background**: Evidence linking chlorinated polyfluoroalkyl ether sulfonic acids (Cl-PFESAs) to preterm birth (PTB) is limited, and their relationships with the metabolome remain unexplored. **Aims:** Our study aimed to explore the role of the metabolome in the associations between Cl-PFESAs exposure and PTB. **Methods**: We conducted a nested case–control study in the Shenyang birth cohort, which included 206 spontaneous preterm birth cases and 206 full-term controls, matched for maternal age and pre-pregnancy BMI. We used conditional logistic regression models to analyze the associations between Cl-PFESAs exposure in umbilical cord blood and PTB. Moreover, the metabolomics of maternal blood (44 cases) between the preterm and control groups was analyzed using the interaction analysis. **Results**: We observed that a higher natural log-transformed 6:2 Cl-PFESA level was associated with greater odds of PTB in conditional multivariable-adjusted logistic regression models (OR = 1.738, 95% CI: 1.320, 2.287). The results of metabolomics pathway analysis showed that histidine metabolism pathways may modify the above risk. When stratified by histidine levels, the association between cord blood 6:2 Cl-PFESA and PTB was different. **Conclusions**: Intrauterine exposure to 6:2 Cl-PFESA was associated with increased PTB. Also, for the first time, our study illustrates that maternal plasma metabolite profiles may modify the associations of 6:2 Cl-PFESA with PTB. More research is needed to elucidate the mechanism underlying the reproductive toxicity of 6:2 Cl-PFESA in pregnant women following exposure.

## 1. Introduction

Preterm birth (PTB, defined as delivery before 37 gestational weeks) affects approximately 15 million neonates globally each year and is a major contributor to infant morbidity and mortality [1]. The prevalence of PTB has been steadily increasing in most countries in recent decades, accounting for 5–18% of all newborns globally and 10–11% in the United States [2,3,4]. Preterm infants are at increased risk of various health complications, such as respiratory and kidney diseases, neurodevelopmental disorders, and cognitive dysfunction [5,6,7], coupled with a substantial economic burden on families and society [8]. Therefore, understanding the etiology of PTB is critical to developing effective preventive strategies and improving the long-term health outcomes of this vulnerable population. Increasing evidence shows that environmental pollutants, especially environmental endocrine-disrupting chemicals, are closely related to the risk of preterm birth [9,10]. Furthermore, the initiation of labor is a complex process involving hormonal, immunological, and metabolic changes [11]. However, the complex interplay between environmental chemical exposure, maternal metabolism, and preterm birth has not been fully elucidated.

Evidence is accumulating regarding the effects of poly- and perfluoroalkyl substances (PFASs) on birth outcomes. Due to their thermal and chemical stability, these compounds are widely used in the manufacture of waterproof, oil-proof, and heat-resistant materials and food packaging [12,13]. Hence, the effects of PFAS on health are an increasing concern [14,15,16]. Both perfluorooctane sulfonate (PFOS) and perfluorooctanoic acid (PFOA), two legacy PFAS, are classified as persistent organic pollutants (POPs) by the Stockholm Convention [17]. Despite this, these compounds are still essential in manufacturing many common products. As a result, efforts have shifted toward developing and adopting safer alternatives.

Chlorinated polyfluorinated ether sulfonic acids (Cl-PFESAs) are the alternatives to PFOS with a similar chemical structure. They have been widely used in the Chinese electroplating industry. Cl-PFESAs have the trade name F-53B and consist of 6:2 Cl-PFESA, which is the main component, and 8:2 Cl-PFESA [18]. Studies have documented the presence of Cl-PFESAs in indoor dust, atmosphere, soil–plant systems, and human serum [19,20]. Notably, 6:2 Cl-PFESA has stronger bioaccumulation and durability than PFOS [21]. Previous literature has shown that 6:2 Cl-PFESA is significantly associated with reproductive and developmental toxicities. It can enter the umbilical cord blood through the placental barrier and has exhibited a higher placental transfer efficiency than PFOS [22,23,24]. Additionally, animal studies have shown that Cl-PFESAs may increase the incidence of PTB [25]. However, epidemiological studies are scarce and have produced inconsistent results. For example, a cohort study in Guangzhou, China, found that maternal serum Cl-PFESA levels were significantly positively associated with PTB [26]. In contrast, another study in Shanxi, China, did not find a significant association between maternal plasma 6:2 Cl-PFESA levels and spontaneous PTB [27]. Therefore, the relationship between Cl-PFESAs exposure and PTB requires further exploration. Given that PFAS exposure is known to induce metabolic disorders [28], comparative metabolomic profiling of full-term and preterm pregnancies could elucidate underlying mechanisms and inform preventive strategies.

Above all, this study aimed to examine the associations between Cl-PFESAs exposure in umbilical cord blood and the incidence of PTB. Additionally, we hypothesized that the potential effects of Cl-PFESAs on PTB might be modified through alterations in the maternal blood metabolite profile. To test this, we sought to identify specific metabolic pathways that could link Cl-PFESAs exposure to PTB.

## 2. Materials and Methods

### 2.1. Population

This study utilized data from the Shenyang birth cohort, based at the Shenyang Women’s and Children’s Hospital in Liaoning, China, between January 2019 and December 2021. This cohort was established to investigate the effects of early-life environmental pollutants exposure on adverse birth outcomes and children’s growth and development. The hospital accounts for approximately one-quarter of all annual births in the Shenyang area. Participants were enrolled in the study at the first time of their prenatal visit. This study recruited 2514 pregnant women aged 18–45 years without infectious diseases and living locally for more than 2 years. Among them, 2429 pregnant women completed a face-to-face questionnaire interview and provided maternal blood samples before delivery and cord blood samples. There were 231 spontaneous PTBs (9.5%), of which 206 were included in the current study after excluding multiple pregnancies (*n* = 25). For each woman with a PTB, a control woman who delivered at full-term was matched by maternal age and pre-pregnancy BMI using the propensity score matching method. In total, 412 pregnant women were included in this study (Figure S1). This study was approved by the Research Ethics Committee of Sun Yat-Sen University, and we collected written informed consent from all participants (Approval: Number 2019010).

### 2.2. Birth Outcomes

Gestational age was calculated by obstetricians based on self-reported time of last menstrual period combined with results of the first-trimester ultrasound exam. PTB was defined as gestational age less than 37 weeks. PTB was further subcategorized by gestational age as follows: (1) late PTB: 34 to <37 weeks, (2) moderate PTB: 32 to <34 weeks, and (3) very PTB: 28 to <32 weeks. Furthermore, based on the reason for occurrence, PTB was also classified as spontaneous or therapeutic.

### 2.3. PFAS Measurements

We collected 5 mL of umbilical cord blood and centrifuged it at 3500 rpm for 5 min, within 2 h of blood collection. After centrifugation, the supernate was dispensed into sterile tubes and immediately stored at −80 °C until analysis. We measured umbilical cord serum for PFAS concentration using a modified version of the method of Benskin et al. [29]. Detailed analysis methods, including standards, reagents, and quality control, have been published previously [26]. It is also presented in the appendix of this study. Briefly, 0.2 mL serum was extracted with 2 mL of 0.1 M formic acid, and solid phase extraction was performed. The detection, separation, and quantification of PFAS were conducted using an Agilent 1290 series ultra-performance liquid chromatography system coupled with an Agilent 6495B triple quadrupole mass spectrometer (Agilent Technologies, Santa Clara, CA, USA). The limit of detection (LOD) for each compound was defined as the concentration of each peak analytical level required to achieve a signal-to-noise ratio of 3:1. We replaced all serum PFAS concentrations less than the LOD with LOD divided by the square root of 2. We included PFOS and PFOA, which are the most common legacy PFAS, and 6:2 Cl-PFESA and 8:2 Cl-PFESA, the two alternatives, in our analysis.

### 2.4. Metabolomics Analysis

A total of 44 women from the overall case–control data were included in the maternal blood metabolomics analysis in late pregnancy, including 11 cases and 33 controls matched by age and pre-pregnancy BMI. We evaluated the representativeness of the metabolomics subset.

We used ultra-performance liquid chromatography-quadrupole time-of-flight mass spectrometry (UHPLC-QTOF-MS, Agilent, Santa Clara, CA, USA) for metabolomics analysis of maternal plasma. Chromatographic separation was conducted using a C18 reversed-phase column (2.1 mm × 100 mm, 1.7 μm), and the column temperature was at 40 °C. The separation was achieved using a C18 column (2.1 mm × 100 mm, 1.7 μm). Mobile phase A was 0.1% formic acid aqueous solution, and mobile phase B was 0.1% formic acid acetonitrile solution. The injection volume was 5 μL, and the flow rate was 0.3 mL/min. We used an electrospray ionization source to perform mass spectrometry, with data collected in positive and negative ion modes. The parameters were set as capillary voltage of 3.0 kV (positive ion mode) or 2.5 kV (negative ion mode) and ion source temperature of 150 °C. The mass spectrometry scanning range was *m*/*z* 50–1200, and the scanning time was 0.2 s. All samples were analyzed in randomized order across analytical batches. To ensure data reliability, quality control (QC) samples were inserted prior to the analysis of each batch of samples. QC samples were inserted after every 10 samples to monitor retention time, peak intensity, and stability of the mass spectral response during analysis.

Metabolite data were normalized using probabilistic quotient normalization (PQN) and subsequently log 10-transformed to approximate a normal distribution prior to statistical analysis. Metabolic pathway enrichment analysis was conducted using the MetaboAnalyst 6.0 platform (https://dev.metaboanalyst.ca/). Screen key metabolic pathways associated with pregnancy outcomes were based on the Kyoto Encyclopedia of Genes and Genomes (KEGG) database, and their biological significance was assessed by topological analysis. All *p*-values were adjusted for multiple testing using the false discovery rate (FDR) method, with an FDR threshold of <0.10.

### 2.5. Covariates

Potential covariates were selected based on the previous literature [26,27]. These covariates mainly consisted of factors affecting both PFAS levels and PTB outcomes. The final covariates included occupation (blue collar or white collar), educational attainment (≤high school or >high school), annual household income in Chinese Yuan (RMB) (≤100,000 or >100,000), parity (primipara or multipara), and infant sex (male or female). As the included pregnant women had no smoking or alcohol consumption, these variables were not considered as covariates. In addition, since the study population consisted predominantly of Han Chinese, race was also not included in this analysis.

### 2.6. Statistical Analysis

Demographic data were summarized using descriptive statistics appropriate for their distribution. Categorical variables are presented as frequencies (*n*, %), normally distributed continuous variables as mean ± standard deviation, and non-normally distributed variables as median (interquartile range). Group differences were assessed using chi-square tests for categorical variables, independent *t*-tests for normally distributed continuous data, and Wilcoxon rank-sum tests for non-normally distributed data. To address right-skewness, serum PFAS concentrations were naturally log-transformed prior to analysis.

We examined the association between umbilical cord serum PFAS and PTB outcomes using conditional logistic regression. Moreover, tertiles of PFAS exposure were included in the models, and the lowest tertile was used as the reference. Additionally, we used generalized linear models to examine the association between serum PFAS exposure and gestational age. This association was analyzed separately for the different PTB subtypes: late, moderate, and very PTB. We also conducted analyses stratified by infant sex and assessed potential effect modification by including an interaction term for PFAS and sex.

We detected the differential metabolites between the preterm birth and term birth groups and divided the differential metabolites into two groups based on the median value. We analyzed the correlation between cord serum PFAS and preterm birth risk using logistic regression stratified by the differential metabolites groups and calculated the modifying effect of characteristic metabolites between exposure and outcome by entering the multiplicative interaction term into the models. In addition, causal mediation analyses were used to estimate the mediation effects of metabolites on the associations between PFAS and preterm birth.

Furthermore, we performed several sensitivity analyses. To rule out the possibility of interfering with PTB outcomes, we separately excluded participants with hypertensive disorders in pregnancy (HDP), gestational diabetes mellitus (GDM), and gestational thyroid dysfunction. All data were statistically analyzed using SAS software (version 9.4, SAS Institute Inc., Cary, NC, USA), and all tests were two-tailed and statistically significant when *p* < 0.05 for main effects and *p* < 0.10 for interaction terms [30].

## 3. Results

### 3.1. Characteristics and Exposure Profile of Participants

Table 1 shows the characteristics of the 412 mother–infant pairs. The mean maternal age was 29.54 ± 3.95 years, and the average pre-pregnancy BMI was 22.64 ± 3.75 kg/m^2^. Most participants had attained education beyond high school, had an annual household income below 100,000 yuan, were employed in blue-collar occupations, and were primiparous. Maternal occupation differed significantly between the preterm and full-term groups, while no other demographic characteristics showed notable differences.

In cord blood, PFOA (median: 1.635 ng/mL) was the most abundant PFAS, followed by PFOS (median: 0.886 ng/mL) and 6:2 Cl-PFESA (median: 0.303 ng/mL). As shown in Table S1, the detection rates of PFOS, PFOA, 6:2 Cl-PFESA, and 8:2 Cl-PFESA were 100.0%, 100.0%, 96.0%, and 71.0%, respectively. Additionally, among PFAS components with a detection rate > 85%, only 6:2 Cl-PFESA showed a statistically significant difference in concentration between the two groups, which was significantly higher in the preterm group compared to the full-term group. Spearman correlation coefficients between PFASs ranged from 0.11 to 0.55 (Table S2).

### 3.2. Association Between Umbilical Cord Serum PFAS and PTB

Table 2 shows the odds ratios (OR) and 95% confidence intervals of cord serum PFOS, PFOA, 6:2 Cl-PFESA, and 8:2 Cl-PFESA, with PTB in conditional logistic regression models. The results showed that elevated levels of natural log-transformed 6:2 Cl-PFESA were significantly associated with an increasing risk of PTB after adjusting for covariates (OR = 1.738, 95% CI: 1.320, 2.287). Compared with the lowest tertile of 6:2 Cl-PFESA, the highest tertile was associated with a significantly higher PTB risk (OR = 1.833, 95% CI: 1.134, 2.962, *p* < 0.05). Sex-stratified analyses (Table S3) revealed a more pronounced association in male infants (OR = 1.608, 95% CI: 1.005, 2.574) than in female infants (OR = 1.683, 95% CI: 0.901, 3.143), with a significant interaction effect (*p*_interaction_ = 0.028).

After excluding pregnant women with HDP (*n* = 324), gestational diabetes mellitus (*n* = 148), and gestational thyroid dysfunction (*n* = 374), the results were consistent with the main findings of this study (Table S4).

### 3.3. Association Between Umbilical Cord Serum PFAS and Gestational Weeks

**Figure 1:**
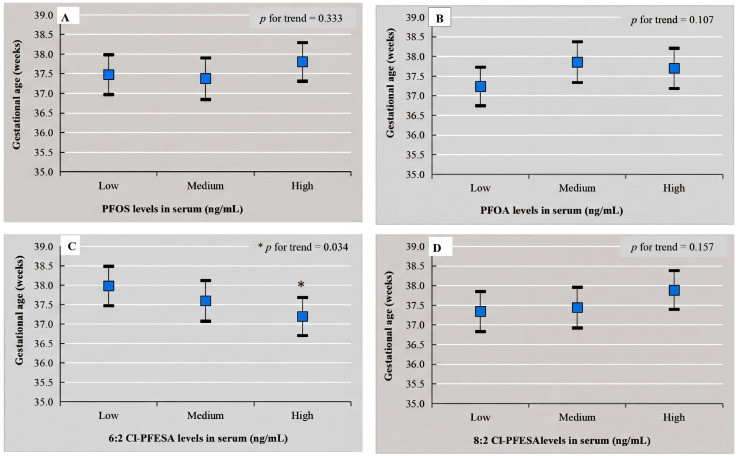
Dose–response relationship between cord serum PFAS tertiles exposure and gestational age ((**A**) PFOS; (**B**) PFOA; (**C**) 6:2 Cl-PFESA; (**D**) 8:2 Cl-PFESA). Abbreviations: Cl-PFESA, chlorinated polyfluorinated ether sulfonic acids; PFOS, perfluorooctane sulfonate; PFOA, perfluorooctanoic acid. Adjusted for maternal occupation, education, annual household income, parity, and infant sex.

Figure 1 describes that the highest tertiles exposed to 6:2 Cl-PFESA had significantly lower gestational age compared with the lowest tertiles, with a trend of *p* < 0.05. As presented in Table S5, when PTB was stratified by different PTB subtypes, serum 6:2 Cl-PFESA was significantly negatively associated with gestational age only in moderate PTB groups (β = −0.442, 95% CI: −0.854, −0.031)**.**

### 3.4. Metabolomics Modify the Association Between 6:2 Cl-PFESA and PTB

Table S6 presents the characteristics of the 44 pregnant women. Maternal blood samples collected during late-term pregnancy were analyzed using metabolomics. Table S7 demonstrates the comparability of the metabolomics subset with the overall cohort in terms of baseline characteristics, supporting its use as a representative sample for analysis. Figure S2 shows the significant associations between umbilical cord serum PFAS concentrations and PTB.

A total of 576 metabolites were screened for differences between the preterm and full-term groups. Figure S3A shows hierarchical clustering analysis of the significantly altered metabolites in maternal plasma. Volcano plot analysis (Figure S3B) further confirmed that a total of 16 metabolites were significantly upregulated in the preterm group, while 15 metabolites were significantly downregulated. Metabolite set enrichment analysis (MSEA) revealed that histidine metabolism, linoleic acid metabolism, and biosynthesis of unsaturated fatty acids were the most significantly enriched pathways (Figure S4A). Pathway impact analysis further indicated that histidine metabolism and linoleic acid metabolism exhibited both high pathway impact and statistical significance (Figure S4B).

**Figure 2:**
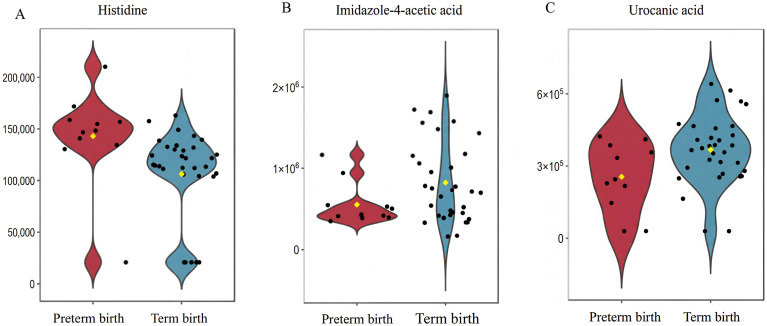
The levels of differential metabolites in the histidine metabolic pathway in the preterm birth (PTB) and term birth groups. Note: (**A**) histidine, (**B**) imidazole-4-acetic acid, and (**C**) urocanic acid. Black dots: Represent individual data points, showing the metabolite concentration of each sample in the preterm birth and term birth groups. Yellow dots: Represent the median value of the metabolite concentration in each group, which is a measure of central tendency for the data distribution.

Overall, the most prominent alteration in the plasma metabolome of women with PTB was the histidine metabolism pathway. Within this pathway, histidine was upregulated, while downstream metabolites, including imidazole-4-acetic acid and urocanic acid, were downregulated (Figure 2).

**Figure 3:**
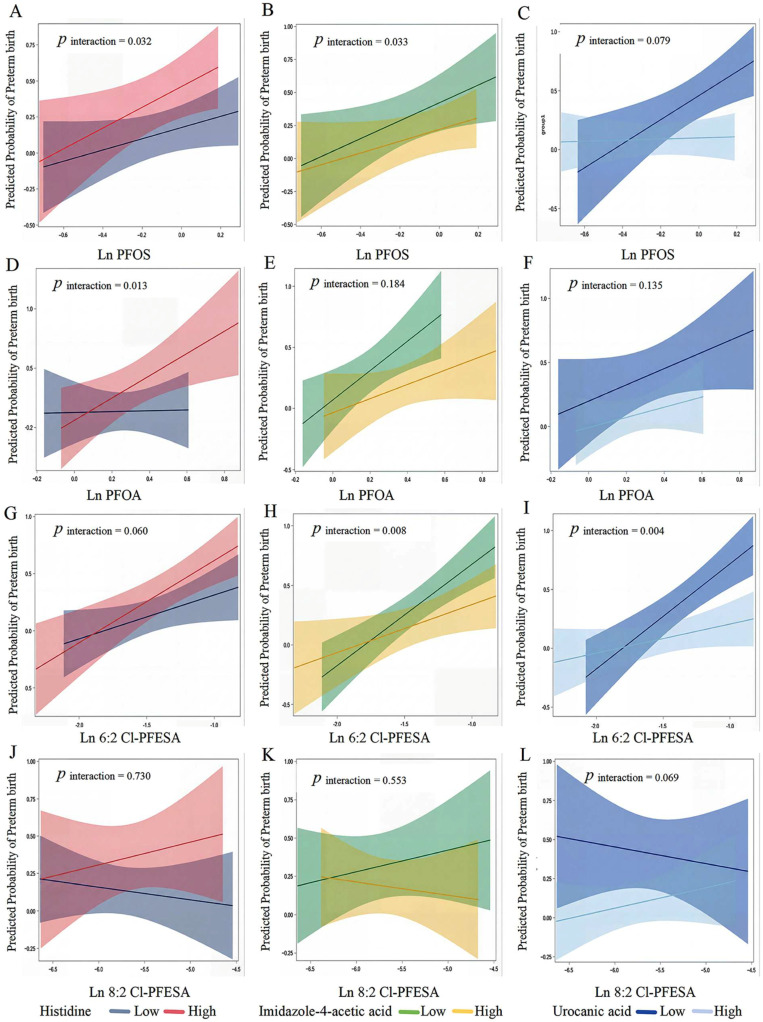
Maternal plasma metabolites modify the associations of umbilical cord serum PFAS (ln ng/mL) with preterm birth risk. Note: Odds ratios (lines) and 95%CIs (shaded) are shown for PFOS (**A**–**C**), PFOA (**D**–**F**), 6:2 Cl-PFESA (**G**–**I**), and 8:2 Cl-PFESA (**J**–**L**) in relation to PTB, stratified by median-dichotomized levels of histidine (**A**,**D**,**G**,**J**), imidazole-4-acetic acid (**B**,**E**,**H**,**K**), and urocanic acid (**C**,**F**,**I**,**L**). *p*_interaction_ < 0.1 was defined as statistical significance.

In an exploratory analysis, we considered that the histidine metabolism pathway may modify the associations between serum PFAS concentrations and PTB (Figure 3). Specifically, maternal histidine levels may interact with umbilical cord serum exposures to PFOA, PFOS, and 6:2 Cl-PFESA in relation to PTB. Moreover, the association between 6:2 Cl-PFESA and PTB may also be modified by imidazole-4-acetic acid and urocanic acid. Stratified analyses may indicate stronger associations between 6:2 Cl-PFESA and PTB among women with higher histidine levels, as well as among those with lower levels of imidazole-4-acetic acid and urocanic acid. We did not observe mediation effects for metabolites in maternal plasma on the association between PFAS and preterm birth (Table S8).

## 4. Discussion

This study provides novel evidence by demonstrating a significant positive association between umbilical cord serum 6:2 Cl-PFESA exposure and an increased risk of PTB. Furthermore, untargeted metabolomic profiling revealed that the plasma metabolome of women who delivered preterm was significantly altered, with the histidine metabolism pathway identified as the most prominently disturbed. Importantly, we established that maternal histidine metabolism may act as an effect modifier, influencing the association between 6:2 Cl-PFESA exposure and PTB. These findings not only highlight 6:2 Cl-PFESA as a potential environmental risk factor for PTB but also uncover a novel metabolic mechanism involving histidine metabolism, offering new insights for future mechanistic research and preventive strategies.

### 4.1. Umbilical Cord Serum Cl-PFESAs Exposure Assay

In this study, the median concentration of 6:2 Cl-PFESA in cord serum was 0.303 ng/mL, with a detection proportion of 96.0%. This finding is consistent with several previous reports from different regions of China. For instance, studies from a Guangzhou birth cohort (*n* = 424, median: 0.32 ng/mL, detection proportion: 99.53%) [22], a Beijing birth cohort (*n* = 53, median: 0.58 ng/mL, detection proportion: 95.98%) [23], and a Shandong provincial cohort (*n* = 326, median: 0.27 ng/mL, detection proportion: 100%) [24] reported comparable concentrations and detection frequencies. A study from Wuhan (*n* = 100) [31] reported a slightly higher concentration (median: 0.80 ng/mL, 100% detection). Collectively, these results indicate that 6:2 Cl-PFESA is widely detectable among fetuses in China, underscoring the need for further investigation into its potential health effects on pregnancy outcomes.

### 4.2. Association Between Cord Serum Cl-PFESAs and PTB

Current evidence on the association between cord blood 6:2 Cl-PFESA and PTB remains limited. To date, only two studies have investigated the relationship between maternal serum 6:2 Cl-PFESA and PTB. A 2021 birth cohort study in Guangzhou, China (*n* = 372), which measured serum 6:2 Cl-PFESA levels at an average gestational age of 33 weeks (median concentration: 2.41 ng/mL), reported a significant positive association with PTB (OR = 2.67, 95% CI: 1.73, 4.15) and a reduction in gestational age by 0.39 weeks per ln-unit increase (95% CI: −0.56, −0.22) [26]. In contrast, a 2020 study from Shanxi Province, China (*n* = 519), that used plasma samples collected earlier in pregnancy (4–22 weeks) with lower exposure levels (median: 0.34 ng/mL) found only isolated significance in the second quartile of 6:2 Cl-PFESA compared to the first quartile and no associations in other quartiles [27]. This discrepancy may be attributed to differences in sampling timing, exposure levels, or population characteristics. Further well-designed studies are urgently needed to clarify these inconsistent findings.

Moreover, we found that the associations between different PFASs and the risk of preterm birth varied in direction and magnitude. Such heterogeneity may be attributed to several factors. First, regarding toxicological mechanisms, compared with traditional PFOS, 6:2 Cl-PFESA has a broader exposure range, stronger bioaccumulation potential, and diverse biological toxicities [20]. Second, in terms of exposure and pharmacokinetics, 6:2 Cl-PFESA exhibits higher placental transfer efficiency than classical PFOS/PFOA [22]. Additionally, placental cells appear more sensitive to emerging PFOS alternatives, which may increase susceptibility to placental dysfunction [32]. Further population-based studies with larger sample sizes and mechanistic studies are warranted to clarify the effect mechanisms of different PFAS compounds.

The biological mechanisms linking 6:2 Cl-PFESA to PTB remain poorly understood. However, experimental studies suggest that Cl-PFESAs can activate the peroxisome proliferator-activated receptor (PPAR) signaling pathway, with even stronger binding affinity and activation potency than PFOS [33]. PPAR activation has been shown to disrupt lipid and glucose metabolism, immune responses, and promote chronic inflammation [34,35], which are all implicated in the pathogenesis of PTB. Furthermore, studies on zebrafish larvae demonstrate that Cl-PFESAs exposure causes developmental toxicity and disrupts thyroid hormone homeostasis [36], which may further increase PTB risk. Further experimental research is needed to elucidate the precise mechanisms underlying the association between Cl-PFESAs exposure and PTB.

### 4.3. Histidine Metabolic Pathway in the Association Between 6:2 Cl-PFESA and PTB

Metabolomics, a high-throughput approach for characterizing systemic metabolic perturbations, has been increasingly employed to elucidate how environmental exposures during pregnancy contribute to adverse outcomes [37,38]. In this study, untargeted metabolomic profiling may indicate the alterations in the histidine metabolism pathway in both women who delivered preterm and those with high 6:2 Cl-PFESA exposure. This finding may suggest that dysregulation of histidine metabolism may represent a key mechanistic link between 6:2 Cl-PFESA exposure and PTB. Similarly, studies have shown that changes in peripheral blood metabolites occur before the onset of preterm cervical ripening [39]. Furthermore, the impact of prenatal exposure to PFAS on the shortening of pregnancy duration is related to eight metabolic pathways and 52 metabolites in newborn dried blood spots. Histidine plays a critical role in fetal development and maternal health during pregnancy, serving as a precursor for histamine, which is a modulator of immune response, uterine contractility, and placental circulation. Aberrant histidine metabolism may therefore contribute to impaired placental function [40], or premature contractions [41], all of which are established pathways to PTB [42]. Additionally, some literature indicates that imidazole-4-acetic acid and urocanic acid are key biomarkers for histidine metabolism [43,44]. Hence, the alteration of the histidine metabolic pathway may play a certain role in the process of premature birth.

Although there is no literature on the mechanisms of 6:2 Cl-PFESA and the histidine metabolic pathway, some previous studies may provide some evidence. Karthikeyan et al. discovered that prenatal exposure to a high level of PFAS is associated with changes in the umbilical cord serum metabolome [45]. Similarly, Liang et al. observed that prenatal exposure to PFOA and PFOS at 6–17 weeks of gestation is associated with the metabolism of histidine [28]. Furthermore, an experimental study [25] reported that F-53B caused metabolic disorders in the mother mice and abnormal metabolic changes during pregnancy, which may have a significant impact on adverse pregnancy outcomes. In the future, more studies on the mechanism by which metabolites mediate the adverse pregnancy outcomes caused by F-53B are needed.

### 4.4. Strength and Limitation

Our study has several strengths. Firstly, all diagnoses, questionnaire administrations, and sample collections were conducted by professional obstetricians and gynecologists through face-to-face interviews following standardized procedures to minimize information and survey biases. Secondly, we adopted a nested case–control study design, which not only inherited the reliable causal inference advantage of cohort studies but also absorbed the efficient and resource-saving advantages of case–control studies. Additionally, we have thoroughly adjusted for the confounding factors, minimizing biases as much as possible and enhancing the reliability of the results.

However, this study’s limitations inevitably remain. Firstly, the sample size used for metabolomics analysis was relatively small. In the future, metabolomics analysis will require a larger sample size from a broader population to robustly validate our results. Secondly, the metabolomics analysis has limitations of non-targeted detection, including insufficient specificity, complex data interpretation, and weak quantitative ability. Moreover, although we employed statistical corrections such as the FDR to control the risk of false positives, the primary aim of this metabolomics study is hypothesis generation rather than confirmation of causal relationships. Therefore, the metabolite markers and pathways reported here require external validation in independent prospective cohorts with diverse populations and analytical platforms. Thirdly, the metabolomics in this study was conducted using maternal blood samples collected at a single time point. However, metabolic changes influencing preterm birth may occur across multiple gestational periods. Future studies should therefore perform serial metabolomic assessments during early- and mid-pregnancy to better understand critical exposure windows. Furthermore, in vivo and in vitro experimental studies are warranted to further explore the mechanism of how the histidine metabolic pathway modifies the relationship between Cl-PFESAs exposure and the risk of preterm birth.

## 5. Conclusions

Our study provides novel epidemiological evidence establishing a significant association between umbilical cord serum 6:2 Cl-PFESA exposure and an increased risk of PTB, highlighting the potential role of this emerging pollutant in adverse pregnancy outcomes. Furthermore, we also observed alterations in histidine metabolism in the maternal plasma metabolome among women with PTB. Importantly, we found preliminary indications suggesting that histidine metabolism may modify the association between 6:2 Cl-PFESA and PTB. These findings underscore the value of combining environmental exposure assessment with high-throughput metabolomics to elucidate biological pathways linked to clinical outcomes. Further mechanistic studies are warranted to validate the role of histidine metabolism, thereby providing critical evidence for future public health interventions and environmental policy decisions.

## Figures and Tables

**Table 1:** Characteristics of the study population.

Characteristics	Total (*n* = 412)	PTB (*n* = 206)	Term Birth (*n* = 206)	*p*
Demographics				
Maternal age (years) ^a^	29.54 ± 3.95	29.60 ± 4.02	29.48 ± 3.89	0.746
Pre-pregnancy BMI (kg/m^2^) ^a^	22.64 ± 3.75	22.84 ± 3.78	22.45 ± 3.73	0.296
Occupation ^b^				
Blue collar	327 (79.37)	174 (84.47)	153 (74.27)	0.015
White collar	85 (20.63)	32 (15.53)	53 (25.73)	
Education ^b^				
≤High school	130 (31.55)	68 (33.01)	62 (30.10)	0.596
>High school	282 (68.45)	138 (66.99)	144 (69.90)	
Annual household income ^b^				
≤100,000 RMB	224 (54.37)	110 (53.40)	114 (55.34)	0.767
>100,000 RMB	188 (45.63)	96 (46.60)	92 (44.66)	
Parity ^b^				
Primipara ^b^	328 (79.61)	162 (78.64)	166 (80.58)	0.714
Multipara ^b^	84 (20.39)	44 (21.36)	40 (19.42)	
Infant sex ^b^				
Male ^b^	232 (56.31)	116 (56.31)	116 (56.31)	1.000
Female ^b^	180 (43.69)	90 (43.69)	90 (43.69)	
PFAS (ng/mL) in cord serum ^c^				
PFOS	0.886 (0.623, 1.308)	0.902 (0.629, 1.325)	0.823 (0.613, 1.305)	0.265
PFOA	1.635 (1.235, 2.273)	1.588 (1.175, 2.425)	1.669 (1.290, 2.182)	0.594
6:2 Cl-PFESA	0.303 (0.207, 0.442)	0.317 (0.225, 0.488)	0.288 (0.185, 0.401)	0.002
8:2 Cl-PFESA	0.004 (0.001, 0.006)	0.003 (0.001, 0.006)	0.004 (0.001, 0.007)	0.025

Abbreviations: Cl-PFESA, chlorinated polyfluorinated ether sulfonic acids; PFOS, perfluorooctane sulfonate; PFOA, perfluorooctanoic acid; BMI, body mass index; PTB, preterm birth; RMB, Chinese Yuan. ^a^ Values are mean ± SD, with differences tested using independent *t*-tests. ^b^ Values are *n* (%), with differences tested using chi-square tests. ^c^ Values are median (quartile1, quartile3), with differences tested using the Wilcoxon rank-sum test.

**Table 2:** Adjusted odds ratios (OR) and 95% confidence intervals for associations of PTB with natural log-transformed cord serum PFAS concentrations (ln ng/mL).

PFAS	PTB OR (95% CI)	
	Crude Model	Adjusted Model ^a^
PFOS		
Per in ln ng/mL greater	1.396 (0.977, 1.996)	1.409 (0.968, 2.050)
Tertile (ng/mL)		
Low (≤0.679)	1.000 (reference)	1.000 (reference)
Medium (>0.679 to 1.134)	1.073 (0.671, 1.718)	1.059 (0.652, 1.720)
High (>1.134)	1.020 (0.646, 1.610)	1.006 (0.626, 1.617)
*p*_for trend_^b^	0.953	0.966
PFOA		
Per in ln ng/mL greater	1.120 (0.753, 1.665)	1.056 (0.703, 1.588)
Tertile (ng/mL)		
Low (≤1.364)	1.000 (reference)	1.000 (reference)
Medium (>1.364 to 1.988)	0.662 (0.416, 1.053)	0.597 (0.369, 0.967)
High (>1.988)	0.781 (0.492, 1.239)	0.685 (0.422, 1.112)
*p*_for trend_^b^	0.216	0.098
6:2 Cl-PFESA		
Per in ln ng/mL greater	**1.677 (1.298, 2.166)**	**1.738 (1.320, 2.287)**
Tertile (ng/mL)		
Low (≤0.237)	1.000 (reference)	1.000 (reference)
Medium (>0.237 to 0.382)	1.331 (0.819, 2.164)	1.317 (0.796, 2.178)
High (>0.382)	**1.772 (1.121, 2.802)**	**1.833 (1.134, 2.962)**
*p*_for trend_^b^	**0.049**	**0.044**
8:2 Cl-PFESA		
Per in ln ng/mL greater	0.836 (0.670, 1.044)	0.809 (0.642, 1.020)
Tertile (ng/mL)		
Low (≤0.002)	1.000 (reference)	1.000 (reference)
Medium (>0.002 to 0.005)	0.587 (0.362, 0.952)	0.610 (0.372, 1.002)
High (>0.005)	0.514 (0.315, 0.838)	0.481 (0.289, 0.801)
*p*_for trend_^b^	0.019	0.016

Abbreviations: CI, confidence interval. ^a^ Adjusted for maternal occupation, education, annual household income, parity, and infant sex. ^b^ Tested using the median value for each category. Note: Bold characters indicate significance, *p*-value < 0.05.

## Data Availability

The data presented in this study are available on request from the corresponding author.

## Supplementary Materials

The following supporting information can be downloaded at: https://www.mdpi.com/article/10.3390/toxics14020142/s1, Table S1. The quantification of PFAS in cord serum. Table S2. Spearman correlation coefficients between serum PFASs analyzed in study participants. Table S3. Adjusted ORs (95% confidence intervals) for preterm birth with natural log-transformed serum PFAS concentrations (ln ng/mL), stratified by infant sex. Table S4. Adjusted ORs (95% confidence intervals) for associations of preterm birth with natural log-transformed cord serum PFAS concentrations (ln ng/mL), excluding participants with hypertensive disorders of pregnancy, gestational diabetes mellitus, and thyroid diseases. Table S5. Adjusted regression coefficients (95% confidence intervals) for associations of gestational weeks of delivery in late preterm birth (34–<37 weeks), moderate preterm birth (32–<34), and very preterm birth groups (28–<32 weeks) with natural log-transformed serum PFAS concentrations (ln ng/mL). Table S6. Characteristics of the population for maternal serum metabolomics analysis. Table S7. Comparison of baseline characteristics of the metabolomics subset and the overall cohort. Table S8. Mediation effects of metabolites on the association between PFAS and preterm birth. Figure S1. Participant flowchart. Figure S2. Metabolomic pathway analysis of differential metabolites in the preterm birth and term birth groups. Figure S3. Transcriptomic expression profile of placental tissues in preterm birth and term birth groups. Figure S4. Scatter plot for associations between preterm birth and natural log-transformed cord serum PFAS concentrations (ln ng/mL). References [29,46] are cited in the Supplementary Materials.
