# Supplementary material for: Histidine Metabolic Pathway Modifies the Relationships Between 6:2 Cl-PFESA Exposure and Preterm Birth
Source: Toxics. 2026 Jan 30;14(2):142. doi: 10.3390/toxics14020142 (PMC12944648; doi:10.3390/toxics14020142)
Supplement: Supplementary file 1 [file toxics-14-00142-s001.zip › toxics-4062636-supplementary.pdf]

Supplementary Materials

Histidine Metabolic Pathway Modifies the Relationships Between 6:2 Cl-PFESA Exposure and Preterm Birth

Table of Contents

PFAS measurement and Quality control

Table S1. The quantification of PFAS in cord serum.

Table S2. Spearman correlation coefficients between serum PFASs analyzed in study participants.

Table S3. Adjusted ORs (95% confidence intervals) for preterm birth with natural log-transformed serum PFAS concentrations (ln ng/mL), stratified by infant sex.

Table S4. Adjusted ORs (95% confidence intervals) for associations of preterm birth with natural log-transformed cord serum PFAS concentrations (ln ng/mL), excluding participants with hypertensive disorders of pregnancy, gestational diabetes mellitus, and thyroid diseases.

Table S5. Adjusted regression coefficients (95% confidence intervals) for associations of gestational weeks of delivery in late preterm birth (34–< 37 weeks), moderate preterm birth (32–< 34), and very preterm birth groups (28–< 32 weeks) with natural log-transformed serum PFAS concentrations (ln ng/mL).

Table S6. Characteristics of the population for maternal serum metabolomics analysis.

Table S7. Comparison of baseline characteristics of the metabolomics subset and the overall cohort.

Table S8. Mediation effects of metabolites on the association between PFAS and preterm birth.

Figure S1. Participant flowchart.

Figure S2. Metabolomic pathway analysis of differential metabolites in the preterm birth and term birth groups.

Figure S3. Transcriptomic expression profile of placental tissues in preterm birth and term birth groups.

Figure S4. Scatter plot for associations between preterm birth and natural log-transformed cord serum PFAS concentrations (ln ng/mL).

PFAS measurement and Quality control

Standards and reagents

All PFAS standards were purchased from Wellington Laboratories (Guelph, ON, Canada). PFAS, including the legacy PFAS, and the alternatives' concentrations in serum samples were detected. Formic acid (High-performance liquid chromatography, HPLC grade, > 98%), ammonium formate (> 99%), and ammonium hydroxide (28.0-30.0%) were purchased from Sigma-Aldrich (Milwaukee, WI). Methanol (HPLC grade) was purchased from Burdick & Jackson (Honeywell International Inc., USA), and acetonitrile (HPLC grade) was purchased from Fischer Scientific (Thermo Fisher Scientific Inc., USA). Distilled water was obtained by Milli-Q (EPED, China).

Sample extraction

The frozen samples could reach room temperature and then were extracted as described previously with minor modifications (Zhang et al., 2013). Briefly, serum was prepared for extraction by mixing 2 mL of 0.1 M formic acid with 0.2 mL of serum. After the addition of 0.5 ng mass-labeled internal standards (0.5 ng of each), the mixture was thoroughly vortexed. The Oasis-HLB cartridge (Waters, 200 mg/6cm^3) was conditioned with 2 mL of methanol (HPLC grade), followed by 2 mL of 0.1 M formic acid. The prepared serum was then loaded into the

column and washed with 3 mL of 0.1 M formic acid, 6 mL of 50% 0.1 M formic acid/50% methanol, and 1 mL of 1% ammonium hydroxide. The cartridge was dried by vacuum. Then, the PFASs were eluted with 2 mL of 1% ammonium hydroxide in acetonitrile. The eluate was evaporated to near-dryness under a gentle stream of high-purity nitrogen at 40 °C and then reconstituted with a mixture of 70 μL of methanol and 30 μL of 20 mM ammonium formate. The extracts were then transferred to a polypropylene centrifuge tube and centrifuged at 12,000 rpm for 10 min at 4 °C. Eighty microliters of the supernatant were transferred to a polypropylene autosampler vial for UPLC-MS/MS analysis.

Liquid chromatography–mass spectrometry

Total PFAS and isomer-specific analyses were performed by UPLC-MS/MS using the method developed by Benskin et al. (Benskin et al., 2012) with a few modifications. Briefly, extracts (5 μL) were injected into a Zorbax Eclipse Plus C18 Column (1.8 μm, 50 mm × 2.1 mm, Agilent Technologies, Santa Clara, CA) equipped with an Ascentis Express F5 PFP guard column (2.7 μm, 30 mm × 2.1 mm, Sigma-Aldrich), both maintained at 30 °C. One Zorbax Eclipse Plus C18 column (5 μm, 50 mm × 4.6 mm, Agilent Technologies, Santa Clara, CA) connected in series was placed directly upstream of the injector to separate PFASs originating from the LC pump from those injected into the analytical column. The mobile phase consisted of 20 mM ammonium formate (solvent A) and 100% methanol (solvent B), maintained at a 0.3 mL/min flow rate. The program started from 55% A and 45% B, which was held for 3 min, ramped to 60% B by 3 min, increased to 63% B by 15 min, and finally to 95% B by 15.5 min, held until 18.5 min, returned to initial conditions by 19 min, and the column equilibrated for a further 4 min. The target analytes were separated and quantified using an Agilent 1290 UPLC attached

to an Agilent 6495B triple-quadrupole tandem MS (Agilent Technologies, Palo Alto, CA, USA) equipped with an electrospray interface operating in negative ion mode. Chromatograms were recorded by multiple reaction monitoring (MRM).

Quality control

PFAS concentrations in the samples were obtained using an internal standards method. Nine calibration curve points were used, between 0.05 and 100 ng/mL, and the coefficient of determination (r2) for each calibration was higher than 0.99. A method blank (calf serum) was extracted with each batch of 22 samples to monitor for any method contamination. We had two kinds of experimental blanks with different matrices in our analysis. One solvent blank (70% methanol/30% water) was injected after every 12 samples to monitor for possible carryover of the instrument. One was a method blank with the commercial calf serum as the matrix to monitor the possible contamination during the extraction procedure. Two quality control standard solutions were run to check the instrumental response and drift, along with 12 samples. The limit of detection (LOD) of each compound was defined as the minimum detectable concentration with a signal-to-noise ratio of 3 (S/N = 3) in a serum sample. The limit of quantification (LOQ) was defined as the concentration of analyte with an S/N ratio of 10. Materials used in the experiment were immersed in methanol for more than 4 h to reduce the background effect. The recovery rates for the target compounds were PFOS: 103.53% ± 10.32%; PFOA: 106.48% ± 10.24%; 6:2 Cl-PFESA: 97.59% ± 13.84%; and 8:2 Cl-PFESA: 85.79% ± 13.85%.

References:

Benskin, J. P., M. G. Ikonomou, M. B. Woudneh and J. R. Cosgrove, 2012. Rapid characterization of perfluoralkyl carboxylate, sulfonate, and sulfonamide isomers by high-performance liquid chromatography-tandem mass spectrometry. J Chromatogr A. 1247. 165-170.

Zhang, Y., S. Beesoon, L. Zhu and J. W. J. E. I. Martin, 2013. Isomers of perfluorooctanesulfonate and perfluorooctanoate and total perfluoroalkyl acids in human serum from two cities in North China. Environ Int. 53(2). 9-17.

Table S1. The quantification of PFAS in umbilical cord serum.

PFASs (ng/mL)	Detection proportion (%)	Median (interquartile range)			*P*
		Total (n = 412)	Preterm birth (n = 206)	Term birth (n = 206)	
PFOS	100.0	0.886 (0.623, 1.308)	0.902 (0.629, 1.325)	0.823 (0.613, 1.305)	0.265
PFOA	100.0	1.635 (1.235, 2.273)	1.588 (1.175, 2.425)	1.669 (1.290, 2.182)	0.594
6:2 Cl-PFESA	95.97	0.303 (0.207, 0.442	0.317 (0.225, 0.488)	0.288 (0.185, 0.401)	0.002
8:2 Cl-PFESA	71.02	0.004 (0.001, 0.006)	0.003 (0.001, 0.006)	0.004 (0.001, 0.007)	0.025
PFHxS	100.0	0.025 (0.013, 0.050)	0.024 (0.010, 0.067)	0.025 (0.015, 0.043)	0.977
PFNA	100.0	0.020 (0.013, 0.030)	0.020 (0.013, 0.030)	0.019 (0.014, 0.030)	0.878
PFDA	100.0	0.146 (0.097, 0.211)	0.146 (0.091, 0.213)	0.146 (0.103, 0.203)	0.566
PFUdA	100.0	0.020 (0.012, 0.033)	0.020 (0.012, 0.033)	0.020 (0.012, 0.033)	0.228
PFTrDA	100.0	0.201 (0.149, 0.269)	0.206 (0.144, 0.274)	0.200 (0.154, 0.264)	0.708
PFHpA	99.42	0.074 (0.053, 0.108)	0.075 (0.049, 0.114)	0.073 (0.056, 0.100)	0.935
PFHpS	98.27	0.104 (0.075, 0.143)	0.102 (0.070, 0.139)	0.107 (0.078, 0.144)	0.874
PFDoA	97.31	0.018 (0.013, 0.027)	0.017 (0.012, 0.026)	0.019 (0.014, 0.028)	0.150
PFTeDA	93.86	0.171 (0.118, 0.254)	0.171 (0.113, 0.259)	0.169 (0.122, 0.248)	0.613
PFHxA	89.64	0.012 (0.008,	0.012 (0.008, 0.020)	0.013 (0.008, 0.019)	0.867
PFPeS	59.31	0.004 (0.002,0.007)	0.003 (0.002, 0.007)	0.004 (0.002, 0.007)	0.099
NEtFOSAA	32.25	0.001 (0.001, 0.002)	0.001 (0.001, 0.002)	0.001 (0.001, 0.002)	0.721
FOSA	30.52	0.001 (0.001, 0.001)	0.001 (0.001, 0.002)	0.001 (0.001, 0.001)	0.002
PFBA	27.64	0.006 (0.006, 0.409)	0.006 (0.006, 0.614)	0.006 (0.006, 0.006)	0.105
NMeFOSAA	16.89	0.003 (0.003, 0.003)	0.003 (0.003, 0.003)	0.003 (0.003, 0.003)	0.047
PFNS	11.32	0.004 (0.004, 0.004)	0.004 (0.004, 0.004)	0.004 (0.004, 0.004)	0.757
PFDS	9.02	0.003 (0.003, 0.003)	0.003 (0.003, 0.003)	0.003 (0.003, 0.003)	0.093
PFPeA	7.87	0.013 (0.013, 0.013)	0.013 (0.013, 0.013)	0.013 (0.013, 0.013)	0.169
PFBS	4.61	0.007 (0.007, 0.007)	0.007 (0.007, 0.007)	0.007 (0.007, 0.007)	0.993
Total PFAS [a]	-	4.777 (3.619, 6.315)	4.788 (3.775, 7.088)	4.757 (3.524, 6.098)	0.161

[a] Total PFAS is the sum of all PFASs.

Table S2. Spearman correlation coefficients between serum PFASs analyzed in study participants.

	PFOS	PFOA	6:2 Cl-PFESA	8:2 Cl-PFESA
PFOS	1	0.52*	0.55*	0.16*
PFOA		1	0.34*	0.11*
6:2 Cl-PFESA			1	0.42*
8:2 Cl-PFESA				1

**P* value <0.05 for the spearman correlations between PFASs.

Table S3. Adjusted OR and 95% confidence intervals of natural log-transformed umbilical cord serum PFAS concentrations (ln ng/mL) with preterm birth, stratified by infant sex.

**PFAS**	**Preterm birth Adjusted OR (95% CI)** [a]		$P_{\text{interaction}}$ [b]
	Male (n=232)	Female (n = 180)	
PFOS	1.557 (0.780, 3.108)	0.883 (0.388, 2.011)	0.518
PFOA	1.312 (0.615, 2.798)	1.180 (0.483, 2.884)	0.902
6:2 Cl-PFESA	**1.608 (1.005, 2.574)**	1.683 (0.901, 3.143)	**0.028**
8:2 Cl-PFESA	0.960 (0.675, 1.365)	0.379 (0.155, 0.928)	0.730

Abbreviations: for PFASs, see Table 1.

[a] Adjusted for maternal occupation, education, annual household income, and parity.

[b] $P_{\text{interaction}}$ was calculated as PFAS × infant sex entered adjusted regression model.

Bold: indicate significance, *P* < 0.10.

Table S4. Adjusted ORs (95% confidence intervals) for associations of preterm birth with natural log-transformed umbilical cord serum PFAS concentrations (ln ng/mL), excluding participants with hypertensive disorders in pregnancy, gestational diabetes mellitus, or thyroid diseases.

PFAS	**Preterm birth Adjusted OR (95% CI)** [a]		
	Excluding hypertensive disorders in pregnancy (n = 324)	Excluding gestational diabetes mellitus (n = 148)	Excluding thyroid diseases (n = 374)
PFOS	1.400 (0.918, 2.134)	1.068 (0.555, 2.055)	1.451 (0.968, 2.176)
PFOA	1.175 (0.746, 1.850)	0.927 (0.485, 1.770)	1.029 (0.664, 1.595)
6:2 Cl-PFESA	**1.731 (1.275, 2.351)**	**1.582 (1.056, 2.370)**	**1.742 (1.299, 2.335)**
8:2 Cl-PFESA	0.832 (0.643, 1.077)	0.838 (0.557, 1.261)	0.798 (0.625, 1.018)

[a] Adjusted for maternal occupation, education, annual household income, parity, and infant sex.

Bold indicates significance, *P* < 0.05.

Table S5. Adjusted regression coefficients (95% confidence intervals) for associations of gestational weeks with natural log-transformed umbilical cord serum PFAS concentrations (ln ng/mL), stratified by preterm birth type.

**PFAS**	**Gestational age (weeks) Adjusted β (95% CI) [a]**		
	Late preterm birth (n = 173)	Moderate preterm birth (n = 21)	Very preterm birth (n = 12)
PFOS	0.207 (-0.010, 0.423)	-0.196 (-0.684, 0.293)	-0.926 (-2.099, 0.247)
PFOA	0.163 (-0.077, 0.403)	-0.017 (-0.338, 0.305)	-0.496 (-1.670, 0.678)
6:2 Cl-PFESA	0.137 (-0.049, 0.322)	**-0.442 (-0.854, -0.031)**	-0.195 (-1.280, 0.890)
8:2 Cl-PFESA	0.036 (-0.083, 0.156)	-0.034 (-0.339, 0.270)	0.1170 (-1.281, 1.514)

[a] Adjusted for maternal occupation, education, annual household income, parity, and infant sex.

Note: Late preterm birth was defined as 34–< 37 weeks, moderate preterm birth was defined as 32–< 34, and very preterm birth was defined as 28–< 32 weeks.

Bold indicates significance, $P < 0.05$.

Table S6. Characteristics of the population for maternal plasma metabolomic analysis.

Characteristics	Total (n = 44)	Preterm birth (n = 11)	Term birth (n = 33)	*P*
**Demographics**				
Maternal age (years) [a]	30.14 ± 3.62	31.27 ± 3.85	29.76 ± 3.52	0.233
Pre-pregnancy BMI (kg/m^2) [a]	22.13 ± 2.58	21.93 ± 2.69	22.20 ± 2.59	0.766
Occupation [b]				
Blue collar	11 (25.00)	2 (18.18)	9 (27.27)	0.701
White collar	33 (75.00)	9 (81.82)	24 (72.73)	
Education [b]				
≤ High school	14 (31.82)	4 (36.36)	10 (30.30)	0.722
> High school	30 (68.18)	7 (63.64)	23 (69.70)	
Annual household income [b]				
≤ 100,000 RMB	24 (54.55)	6 (54.55)	18 (54.55)	1.000
> 100,000 RMB	20 (45.45)	5 (45.45)	15 (45.45)	
Parity [b]				
Primipara	37 (84.09)	10 (90.91)	27 (81.82)	0.659
Multipara	7 (15.91)	1 (9.09)	6 (18.18)	
Infant sex [b]				
Male	22 (50.00)	5 (45.45)	17 (51.52)	1.000
Female	22 (50.00)	6 (54.55)	16 (48.48)	
**PFAS (ng/mL) in maternal serum** [c]				
PFOS	5.250 (3.471, 6.743)	6.772 (5.958, 8.904)	4.498 (3.266, 5.908)	0.006
PFOA	5.467 (2.859, 6.912)	6.612 (5.632, 8.690)	4.772 (2.705, 6.773)	0.151
6:2 Cl-PFESA	0.773 (0.466, 1.139)	1.136 (0.914, 1.567)	0.633 (0.381, 0.960)	0.005
8:2 Cl-PFESA	0.022 (0.015, 0.037)	0.026 (0.017, 0.034)	0.019 (0.013, 0.040)	0.647
**PFAS (ng/mL) in umbilical cord serum** [c]				
PFOS	1.085 (0.670, 1.160)	1.158 (1.104, 1.204)	0.809 (0.623, 1.138)	0.014
PFOA	1.312 (1.129, 1.608)	1.615 (1.603, 1.784)	1.255 (1.105, 1.407)	0.004
6:2 Cl-PFESA	0.249 (0.161, 0.339)	0.367 (0.335, 0.417)	0.229 (0.154, 0.297)	< 0.001
8:2 Cl-PFESA	0.003 (0.002, 0.005)	0.004 (0.002, 0.005)	0.003 (0.002, 0.006)	0.645

Abbreviations: BMI, body mass index; RMB, Chinese Yuan.

[a] Values are mean ± SD. [b] Values are n (%).

[c] Values are median (quartile1, quartile3), with differences tested using the Wilcoxon rank-sum test.

Table S7. Comparison of baseline characteristics of the metabolomics subset and the overall cohort.

Characteristics	Metabolomics subset (n = 44)	Total (n = 412)	*P*
**Demographics**			
Maternal age (years) [a]	30.14 ± 3.62	29.54 ± 3.95	0.307
Pre-pregnancy BMI (kg/m^2) [a]	22.13 ± 2.58	22.64 ± 3.75	0.141
Occupation [b]			0.469
Blue collar	11 (25.00)	327 (79.37)	
White collar	33 (75.00)	85 (20.63)	
Education [b]			0.969
≤ High school	14 (31.82)	130 (31.55)	
> High school	30 (68.18)	282 (68.45)	
Annual household income [b]			0.948
≤ 100,000 RMB	24 (54.55)	224 (54.37)	
> 100,000 RMB	20 (45.45)	188 (45.63)	
Parity [b]			0.512
Primipara	37 (84.09)	328 (79.61)	
Multipara	7 (15.91)	84 (20.39)	
Infant sex [b]			0.402
Male	22 (50.00)	232 (56.31)	
Female	22 (50.00)	180 (43.69)	

Abbreviations: BMI, body mass index; RMB, Chinese Yuan.

[a] Values are mean ± SD.

[b] Values are n (%).

Table S8. Mediation effects of metabolites on the association between PFAS and preterm birth.

	Histidine		Imidazole-4-acetic acid		Urocanic acid	
	$P_{\text{Total Effect}}$	$P_{\text{mediation}}$	$P_{\text{Total Effect}}$	$P_{\text{mediation}}$	$P_{\text{Total Effect}}$	$P_{\text{mediation}}$
PFOS	0.006	0.324	0.010	0.934	0.006	0.420
PFOA	0.024	0.748	0.030	0.186	0.038	0.466
6:2 Cl-PFESA	< 0.001	0.744	< 0.001	0.084	< 0.001	0.220
8:2 Cl-PFESA	0.680	0.994	0.672	0.842	0.678	0.952

Abbreviations: Cl-PFESA, chlorinated polyfluorinated ether sulfonic acids; PFOS, perfluorooctane sulfonate; PFOA, perfluorooctanoic acid.

Note: $P_{\text{Total Effect}}$ indicates the statistical significance of the total effect, comprising the direct effect of PFAS on preterm birth and the indirect effect mediated through metabolites. $P_{\text{mediation}}$ indicates the statistical significance of the mediation effect.

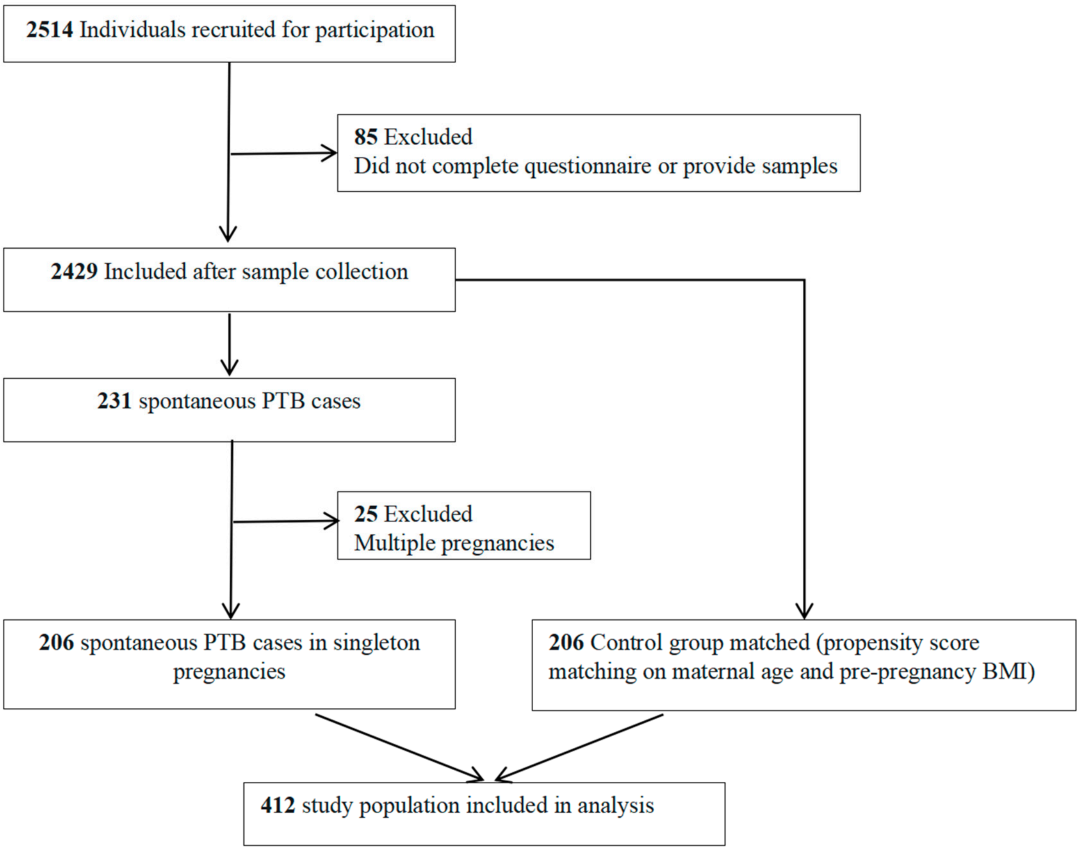

Figure S1. Participant flowchart.

Abbreviations: PTB, preterm birth.

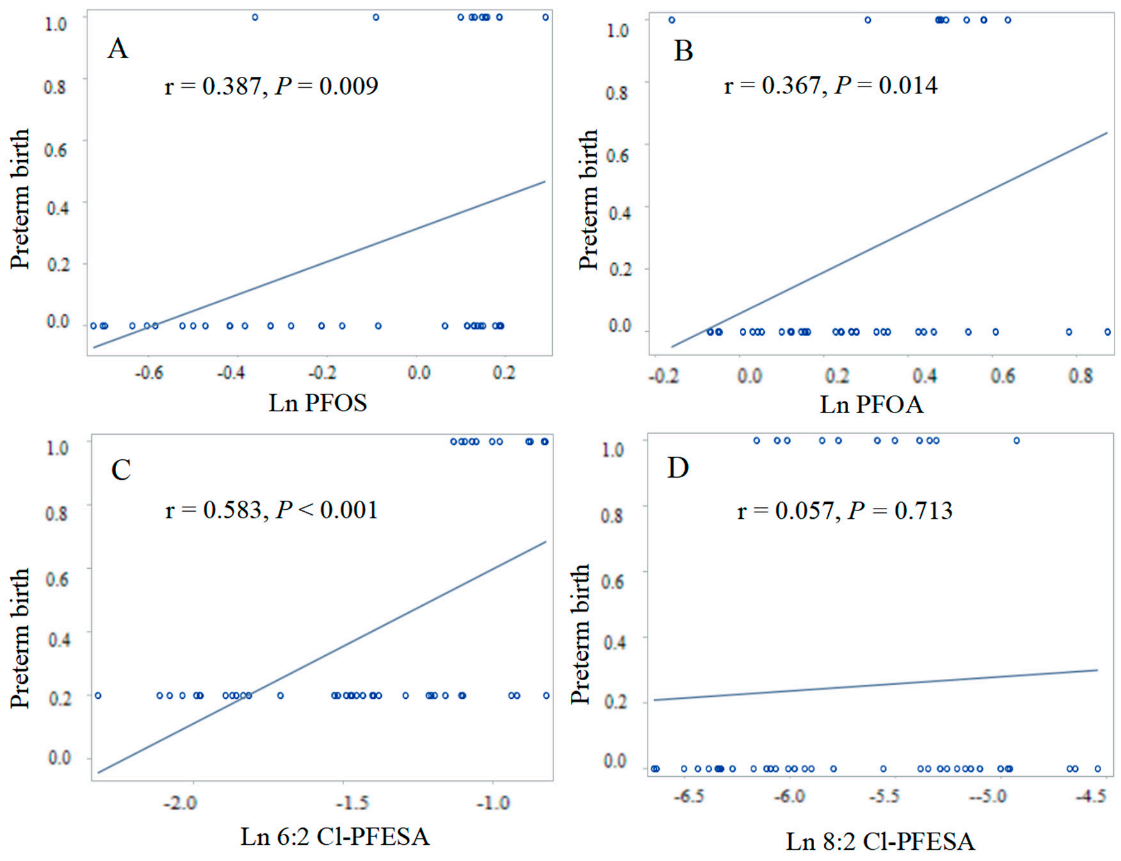

Figure S2. Scatter plot for associations between preterm birth and natural log-transformed cord serum PFAS concentrations (ln ng/mL).

Abbreviations: for PFASs, see Table 1.

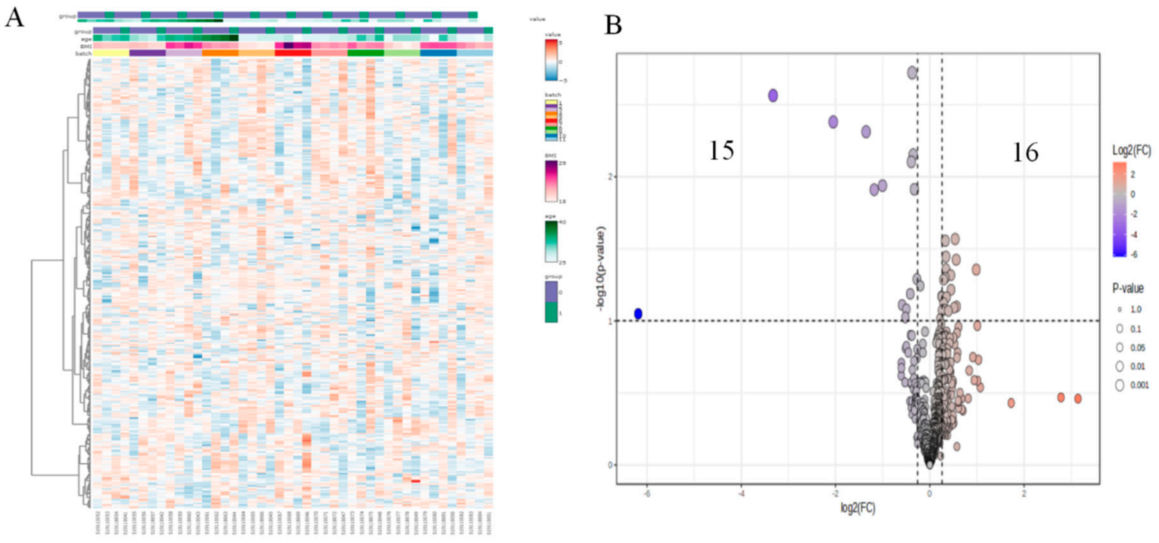

Figure S3. Maternal plasma metabolomics identifies profiles associated with preterm birth.
A: Hierarchical clustering heatmap. B: Volcano plot highlighting significantly upregulated (red) and downregulated (blue) metabolites.

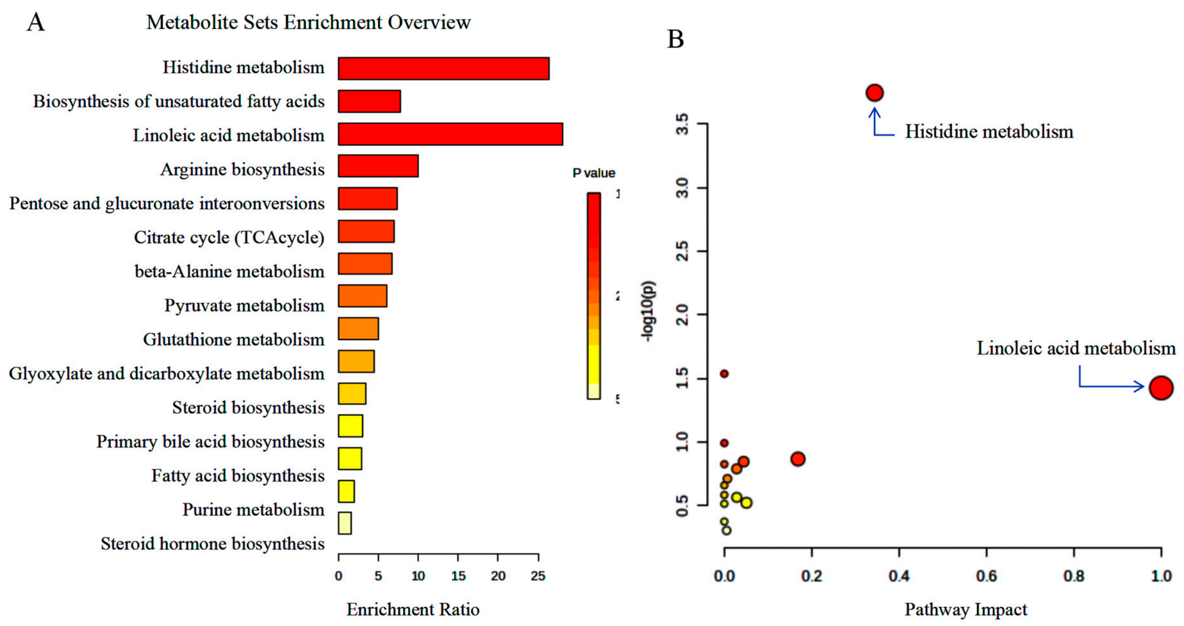

Figure S4. Metabolomic pathway analysis of differential metabolites in the preterm birth and term birth groups.

A: shows the results of the enrichment analysis, of which the different metabolic pathways are sorted by Enrichment Ratio. B: shows the Pathway Impact Analysis. The color of the scattered dots represents the size of the *P*-value, and the size of the dots correlates with the degree of Pathway Impact. The color gradient indicates the significance level of the *P*-value.
